# Comparing theories of consciousness: why it matters and how to do it

**DOI:** 10.1093/nc/niab019

**Published:** 2021-08-18

**Authors:** Simon Hviid Del Pin, Zuzanna Skóra, Kristian Sandberg, Morten Overgaard, Michał Wierzchoń

**Affiliations:** Consciousness Lab, Institute of Psychology, Jagiellonian University, Ingardena 6, Krakow 30-060, Poland; Consciousness Lab, Institute of Psychology, Jagiellonian University, Ingardena 6, Krakow 30-060, Poland; Center for Functionally Integrative Neuroscience, Aarhus Universitet, Universitetsbyen 3, Building 1710, Aarhus C 8000, Denmark; Center for Functionally Integrative Neuroscience, Aarhus Universitet, Universitetsbyen 3, Building 1710, Aarhus C 8000, Denmark; Consciousness Lab, Institute of Psychology, Jagiellonian University, Ingardena 6, Krakow 30-060, Poland

**Keywords:** theories and models, consciousness, theoretical comparison, strong inference

## Abstract

The theoretical landscape of scientific studies of consciousness has flourished. Today, even multiple versions of the same theory are sometimes available. To advance the field, these theories should be directly compared to determine which are better at predicting and explaining empirical data. Systematic inquiries of this sort are seen in many subfields in cognitive psychology and neuroscience, e.g. in working memory. Nonetheless, when we surveyed publications on consciousness research, we found that most focused on a single theory. When ‘comparisons’ happened, they were often verbal and non-systematic. This fact in itself could be a contributing reason for the lack of convergence between theories in consciousness research. In this paper, we focus on how to compare theories of consciousness to ensure that the comparisons are meaningful, e.g. whether their predictions are parallel or contrasting. We evaluate how theories are typically compared in consciousness research and related subdisciplines in cognitive psychology and neuroscience, and we provide an example of our approach. We then examine the different reasons why direct comparisons between theories are rarely seen. One possible explanation is the unique nature of the consciousness phenomenon. We conclude that the field should embrace this uniqueness, and we set out the features that a theory of consciousness should account for.

**Highlights**

- Theories of consciousness differ widely and are developed in isolation.
- Theories should be rigorously compared.
- We define a way in which theories can be assessed and their predictions can be tested experimentally.
- Another way to converge theories is to discuss which aspects they should address.
- These approaches have benefited other fields and may benefit our field despite its unique topic.

## Introduction

In recent decades, the field of scientific studies of consciousness has grown considerably. There have been regular international conferences on the topic, and more empirical results and theoretical works have been published than ever before. Notable contemporary scientific theories of consciousness include *Higher-Order Theories of Consciousness* (Rosenthal 2005; Brown *et al.* 2019), *Global Neuronal Workspace* (GNW) (Dehaene and Changeux 2011), *Integrated Information Theory* (IIT) (Oizumi *et al.* 2014) and *Recurrent Processing (RP)theory* (Lamme and Roelfsema 2000). As a sign of the plurality of theories in the field, several aforementioned theories exist in multiple versions. An example of this is IIT which, like software, is named ‘*IIT 3.0*’ in its most recent formulation. Each theory mentioned here is, to some degree, backed by empirical evidence that aims to confirm its claims. Looking at the impressive number of publications based on this approach, consciousness research seems to be flourishing.

However, some critics claim (see, e.g. Irvine 2014) that the current state of consciousness research is not satisfactory. Irvine notes that even though consciousness researchers use scientific methods, these methods ‘(…) instead of being used to provide independent empirical evidence, and to structure progressive research programs (…) are often used in a post hoc way to support pre-theoretic assumptions, and so contribute to debates only in a superficial way’ (Irvine 2014, 105).

Given Irvine’s critique, one may thus wonder if the plurality of theories and results is actually helpful if the goal is more scientifically accurate descriptions of consciousness. In relation to this issue, Doerig *et al.* (2020) speculate whether the plethora of theories simply reflects a lack of rigour in how we relate theories to empirical results.

To demarcate science, Lakatos (1976) noted that a research programme may be on a path to become pseudoscience if it predicts nothing new or makes predictions that never come to pass. Is this a risk for consciousness studies and, if so, how can we mitigate it?

To address these described shortcomings, we offer a series of steps in this paper that can be taken by individual researchers today. They can potentially also be employed more broadly in the field in the future. The steps include formulating predictions and focusing on more than a single theory. To create an overview, we will first define an approach to comparing multiple theories formally. For comparisons of theories, we discuss some guidelines that we consider beneficial. We then examine whether and how current research papers compare theories in the consciousness field and determine if we can learn anything from an approach currently seen in working memory (WM) studies, which is another area of cognitive psychology. Finally, we consider if WM and consciousness studies have enough similarities that some solutions can be applied in our area.

### Comparisons of multiple theories

The risk related to researchers focussing on just one theory was described by Chamberlin (1897) more than a century ago:

‘The moment one has offered an original explanation for a phenomenon which seems satisfactory, that moment affection for [one’s] intellectual child springs into existence, and as the explanation grows into a definite theory [one’s] parental affections cluster about [the] offspring and it grows more and more dear […] There springs up also unwittingly a pressing of the theory to make it fit the facts and a pressing of the facts to make them fit the theory’. (840)

It can indeed be reasonable to modify theories explicitly so that they fit new observations. However, we take Chamberlin’s assessment to be similar to Irvine’s previously mentioned concerns: a theory, once changed in a post-hoc manner, misleadingly risks stating that it has always predicted the observations at hand or that the observations at least do not pose any threats to the theory.

Chamberlin (1897) believed that the antidote to attachment to a single theory was to create multiple hypotheses. However, he noted that there are costs related to formulating and entertaining them. A formal approach coined *stronginference* (SI) was later proposed by John Platt (1964). SI can be viewed as the first approximation of a method that aims to operationalize a theory, test it and compare it to alternative views. The programme consists of four steps, which were slightly revised by Alger (2019):

1. Conceive of multiple alternative hypotheses to account for the phenomenon you are studying.
2. Devise crucial experiments to exclude one or more of the alternatives.
3. Carry out the experiments and interpret the data.
4. Recycle the procedure to develop subhypotheses and sequential hypotheses to refine the results with further testing (46).

Platt believed that this method is the most efficient way to achieve scientific progress. To illustrate this claim, he quotes a speech by Leo Szilard (central in both the Manhattan Project and the cloning of the first human cell) from a conference about how proteins are synthesized:

‘If you do stupid experiments, and finish one a year, it can take 50 years. But if you stop doing experiments for a little while and think how proteins can possibly be synthesized, there are only about 5 different ways, not 50! And it will take only a few experiments to distinguish these (Szilard 1958, as cited in Platt 1964, 348)’.

SI was thus thought to be an economical approach to formulating a theoretical position and sharpening researchers’ experimental designs. Platt even claimed that fields that often employ SI, such as molecular biology and high-energy physics, progress faster than fields that rarely use this programme. Platt mentions the molecular biology research of Francis Crick [who became a pioneer in investigating the neural correlates of consciousness (NCC)] as an example of a well-performed, logic-driven approach. Crick followed logic-based reasoning in which he systematically devised crucial tests to rule out alternatives step by step (Platt 1964).

Although never using these terms, Platt seems to prefer *theory-driven* rather than *data-driven* research approaches. In his view, equations are useful for making formal definitions. Still, he insisted that logic must come first: the most robust theories can be explained in terms of logic and only then formalized mathematically. However, Platt acknowledges that empirical work more often consists of collecting data that are later analysed and related to theories. Arguably, this approach is even more prevalent today with big data and similar data-driven methods. While such approaches have their merits, he generally sees them as a substitution of causation to correlation. In Platt’s view, data-driven approaches also risk reducing the researcher from a critical thinker to a person who simply collects data.

### Critiques of strong inference

SI has since been critiqued. Some have questioned the historical veracity of Platt’s claims, by pointing out that even great scientists such as Newton also conducted more exploratory research (O’Donohue and Buchanan 2001). Exploratory phases may thus be more relevant than Platt initially acknowledged. In fact, there have been attempts to expand on SI with *Strong Inference Plus* (Jewett 2005), which adds additional steps: an exploratory phase in which hypotheses are generated and a pilot phase to investigate statistical power.

Another critique points to the *Duhem–Quine thesis*, which states that all falsifications are necessarily ambiguous and rely on background assumptions (O’Donohue and Buchanan 2001). Imagine this hypothesis: *the boiling point of water is 100 degrees Celsius*. Is the hypothesis disproved if someone observes water boiling at 90 degrees Celsius? Most would say ‘no’ and check the auxiliary conditions: how high above sea level was the person and how reliable is their thermometer? Even more fundamentally, how do thermometers relate to heat? According to this view, it is thus logically impossible to formulate a crucial experiment that directly falsifies a theory.

While relevant, these critiques seem mitigated once one sees SI as an important tool but not the only one. Chamberlin (1897) noted more than a century ago that it is rash to assume that any single method is *the method* in science. Considering the strengths and weaknesses of SI, we propose moving forward by formulating multiple competing hypotheses based on opposing theories. The goal of such an endeavour should not be to dismiss theories that do not get any support; the aim is to create clarity in the field by highlighting theories that make correct predictions and possibly offer areas of improvement for those that do not. In some ways, this endeavour can achieve benefits similar to *adversarial collaborations* in which two or more researchers with opposing theoretical views agree on an experimental paradigm that can satisfyingly distinguish their predictions.

An adversarial collaboration that shares similarities with SI was recently finalized in the field of WM (Cowan *et al.* 2020). In this paper, the contributors to this collaboration describe the advantages and pitfalls of this method. Even though the collaboration did not lead to any of the senior researchers abandoning their favourite theoretical views, in their opinion it markedly improved the accuracy and clarity of their theories (Cowan *et al.* 2020).

Having laid out a general framework for comparing theories, we examine whether the SI approach has been used in consciousness studies and how theory comparisons have generally been performed in the field, in the following sections.

### Comparisons in consciousness research—a general picture

To illustrate how often SI or similar formal comparisons of multiple hypotheses are performed in consciousness research, we searched for articles mentioning arguably the two theories of consciousness that are currently most prevalent: IIT and GNW (Michel *et al.* 2018). We chose GNW and IIT as they are similar in scope and influence, even though they differ in multiple ways. GNW was developed by enriching a cognitive theory [Global Workspace (GW) theory—Baars and Franklin 2003] with neuro-computational models. These models lead to empirically testable predictions (Dehaene *et al.* 2011). On the other hand, IIT was developed based on phenomenological axioms. These axioms are claimed to lead to inferences about the physical properties of consciousness and testable predictions (Tononi 2008). Despite their different theoretical assumptions, starting points (phenomenological axioms vs. neuro-computational models), postulated NCC or level of formalization, it may turn out that these theories complement rather than compete against each other [as mentioned in Northoff and Lamme (2020) that we will describe in more detail later on]. Especially if given a chance to directly compare them on empirical grounds. For example, increased complexity should follow the activation of the GW at least to some extent. Thus, one may expect that multiple papers will aim to compare the two theories. This led us to wonder how often papers would mention both theories rather than one of them.

**Figure 1.**
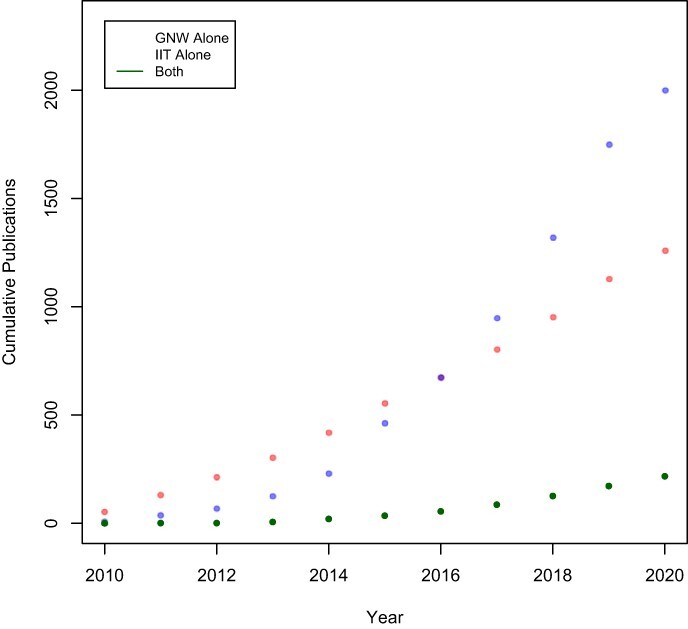
Cumulative publications from 2010 to 2020 matching either or both of the queries ‘GNW’ and ‘IIT’. A total of 1369 and 2210 publications were returned for GNW and IIT, respectively. 251 publications were returned that matched both queries. In the plot, articles that contained both queries have been removed from the GNW and IIT results, so they only indicate papers that did not mention the other theory. We conducted the search on Google Scholar on the 3rd of January 2021

Over the last decade, there has been an impressive increase of scientific publications mentioning either theory, but less than 7% of publications mention both (Fig. 1). At just above 7%, this rate was similar for 2020 alone. Note that this survey does not rule out papers that compared IIT or GNW to yet another theory. It also does not take into account whether a paper offers empirical theory comparison, simply mentions both theories in the context of data interpretation or discusses their assumptions on only a theoretical level. The low proportion is nevertheless striking as it includes mere mentions of these two theories. Therefore, the number of formal comparisons will necessarily be substantially lower. We surveyed the top 10% of the papers deemed most ‘relevant’ according to Google Scholar’s ranking that included both theories. We found that most of them did not compare these theories using multiple hypotheses. When ‘comparisons’ happened, they were often verbal, non-systematic and post hoc, rather than being based on explicit, testable hypotheses, as recommended by SI. A recent paper by Doerig *et al.* (2020, 2) reached a similar conclusion: ‘*Currently, there are very few mutual comparisons between ToCs [Theories of consciousness]. Instead, authors build on their own ToCs, largely ignoring others’.* Sadly, this fits Irvine’s characterization of the field that was mentioned in the introduction. Nevertheless, outside of the criteria used for the survey, we found a few positive examples where theories of consciousness were compared more rigorously.

### Comparisons in consciousness research—examples

There are already papers in the field that aim to compare theories of consciousness. We describe them only briefly, focusing on their general approach to theory comparison. The first two examples represent comparisons, which rely on existing data. In contrast, the latter examples show systematic comparisons, some of which make predictions on novel data.

Boly *et al.* (2017) and Odegaard *et al.* (2017) both reviewed a similar set of evidence relating to the position of the neural correlate of consciousness. They reached opposite conclusions and thereby illustrated a crucial problem with data-driven approaches. A formal comparison of the predictions of either viewpoint before an investigation of the data might have alleviated this outcome. Fleming and Daw (2017) conducted such a formal comparison. They mathematically defined three models based on competing theoretical viewpoints. Next, they derived several predictions based on their preferred model, which they confirmed with previously published data. A benefit of such an approach is that whenever the models disagreed on empirical results, there was an opportunity to learn something rather than confirm or reject a single prediction. However, knowing the results in advance could have biased the formulation of their preferred theory.

The above shortcoming has been addressed by Maniscalco and Lau (2016), who first formulated mathematical models based on three theoretical views and then collected experimental data. Using statistical tests to evaluate the data against the models, they addressed an important point: many models would likely have performed better than a null model if they had been tested individually. Testing models against each is in the spirit of SI while still being data-driven. Similarly, King *et al.* (2016) attempted to formulate theories of consciousness mathematically and compare their predictions systematically. Interestingly, this method appears to follow the outline of SI, although they never refer to it by name.

Finally, we consider the approach by Northoff and Lamme (2020) close to the first steps of both SI and the approach we present in this paper. By systematically reviewing and comparing select theories in the field, they investigated if some theories are complementary rather than competitive. They considered a range of aspects, such as the major areas of interest, typical measures and proposed NCCs. In this comparison, they found that some theories may be compatible as they tackle different aspects of consciousness (e.g. state rather than content, stimulus-related or not). We consider this paper, and the approach itself, as a solid background, to propose empirical predictions and design experiments that could provide evidence in favour or against selected theories.

Based on the examples above, we see that there are attempts at more formal theory comparisons in the field of consciousness, whether this is consciousness defined broadly (King *et al.* 2016; Boly *et al.* 2017; Odegaard *et al.* 2017) or more narrowly (Maniscalco and Lau 2016; Fleming and Daw 2017). More recently, researchers introduced much more general comparisons, consisting of comparisons of multiple consciousness theories (Northoff and Lamme 2020) or proposing common ground to compare theories of interest (Doerig *et al.* 2020). We will lay out the approach we took in the next sections when comparing theories in line with SI. That is, a comparison of verbal theories that rely on performing a crucial test to distinguish between opposing predictions.

### The logic and results from our comparison of theories

In a recently published paper (Del Pin *et al.* 2020), we tested two hypotheses based on the *Partial Awareness Hypothesis* (PAH) (Kouider *et al.* 2010) and RP. Concisely, the PAH states that participants experience complex scenes in a fragmented way, often unnoticed as cues can allow participants to reconstruct scenes. Specifically, the PAH extends GWT to account for graded (phenomenal) accessibility. Based on PAH, we assume that a presentation of a cue that does not contain the same amount of details would reduce performance. For example, if a part of a scene were a red car but only a glimpse of the colour were perceived, the accuracy of its recognition would be different if the cue was the original image than if it were the word ‘car’. RP, however, states that complex scenes are perceived holistically. If this is the case, the cue type should not matter, and the word ‘car’ should be sufficient in our example. In two experiments, we compared the influence of words and images as cues in the partial change report paradigm; we found that the cue type did not influence accuracy.

We thus had two alternative hypotheses:

1. PAH—Accuracy in the image cue condition > accuracy in the word cue condition.
2. RP—Accuracy in the image cue condition = accuracy in the word cue condition.

We could state the experimental logic as a modus tollens argument:

1. *If participants reconstruct objects from cues, then words (which afford less reconstruction) will lead to worse objective performance.*
2. *We observed that words and images had similar objective rates of accuracy.*

*Therefore, participants do not reconstruct objects from cues.*

One can accept this logic without accepting the premises. First, we never demonstrated that words allow less reconstruction than images. Second, we collected moderate Bayesian evidence for the accuracy rates of words and images being the same. It is possible, however, that reconstruction is reduced with word cues, but our paradigm was too coarse to reveal it. Third, even though we replicated our results, there could be something particular about our lab that will prove to be not generalisable. In line with the *Duhem–Quine thesis,* it can thus be argued that our experiment falsified no theory definitively. Still, our results strengthened the RP theory and revealed issues for PAH to address. In the following paragraphs, we will argue that our study provides a background for the formulation of generalisable guidelines for comparing theories.

### The anatomy of our theoretical comparison

Asking good questions is acknowledged in SI, but this approach does not provide explicit guidelines. It may, thus, be valuable to flesh out how we reached the opposing hypotheses. We began by focusing on fundamental questions that both theories may answer. Our goal was to find common ground and identify whether, within that ground, we could formulate diverging predictions. We created Table 1 based on our analysis of the two theories.

**Table 1.** Similarities and differences between two classes of theories of consciousness compared by Del Pin *et al.* (2020). There are considerable overlaps between what the PAH and the RP theory are addressing. We have also found an overlap in paradigms used in studies supporting both approaches, making it more straightforward to formulate diverging predictions

	Prior-driven theories of consciousness	Externally driven theories of consciousness
Theory	PAH	RP theory
Scope	The content of consciousness	The content of consciousness
	The richness of experience	The richness of experience
Positions		
Richness	Sparse. Experience can APPEAR rich, but much is constructed from priors	Experience IS rich, filled with information from the external world
Hierarchy arrangement	Hierarchy of representational levels	One level of conscious representation, but different stages leading to reportability
Binding	Central for the theory	Consciousness is not necessary for visual feature binding
Neural mechanism	Broadcasting information to the frontoparietal network	(Widespread) RP
Temporal dimension	Late (P3b)	Early (VAN)
Attention	Important	Unnecessary
Basis for positions		
Paradigm types	Stimuli presented at threshold and contrastive analysis, e.g. (modified) Sperling task and partial change report paradigm	Stimuli presented at threshold and contrastive analysis, e.g. Sperling task and partial change report paradigm
Behaviour	Objective accuracy	Objective accuracy
Visual masking	Often employed	Thought to disturb unreported weak experiences

After creating an overview of the two theories, we went through a series of steps that can be generalized. These steps may allow others to select theories and formulate opposing hypotheses:

#### Step 1. Define the theory scope

When performing theoretical comparisons, one should select theories that address the same research problem. For example, it is hard to conduct a meaningful comparison of two theories if one addresses the contents of consciousness, whereas the other relates to states. In our approach, we compared two theories that address the contents of consciousness and consider the mechanism behind the richness of experience.

#### Step 2. Check whether the theories’ assumptions are not contradictory

Theories addressing the same research problem might start from contradictory assumptions, which can render a comparison useless to some extent. For example, suppose you wish to compare IIT with another theory. In that case, both should agree with, or at least be able to accept, the axioms of IIT relevant to the comparison. If the other theory claimed that consciousness is not unified, which directly contradicts one of IIT’s axioms, the comparison could be disputable. Consider, therefore, if contradictory assumptions could affect the interpretation of the same empirical evidence.

#### Step 3. Define an essential position on which the theories disagree

If you want to pit two theories against each other, there must be a point of disagreement. This seems crucial in order to propose a paradigm that would allow us to disentangle the compared theories. In our case, RP assumes that experience is rich and filled with information from the external world. On the contrary, PAH assumes that experience appears rich but much is constructed from priors (expectations). This step requires the identification of an effect or a feature that is essential for understanding consciousness. We devote the later section on finding criteria for consciousness theories to this topic.

#### Step 4. Propose an operationalization

Formulate an operationalization that can test the essential position, i.e. a paradigm that allows model comparison. If possible, look for overlaps or similarities between paradigms that are typically used to empirically support either theory empirically. In our case, both the RP and PAH utilized the partial change report task. Different experimental manipulations within the same task, however, supported opposing hypotheses.

#### Step 5. Propose a critical test to measure opposing hypotheses

Once one has a shared paradigm, one needs to define a critical test to compare the theories. In our example, if the performance in a partial change report task is based on rich representations, the type of cue presented should not matter for the task performance. Thus, we assume that if RP is correct, it should not matter whether we cue with a word or an image. If the performance in a partial change report task is based on a reconstruction of the external world, the image cue should be more effective than the word cue in fostering the reconstruction of the original image.

#### Step 6. Use results to infer which hypothesis has gained support

If the critical test is well designed, the result allows us to judge the competing hypotheses. In our case, the results show no difference between the cue types. Thus, RP seems to be more in line with the results than PAH.

When going through the above steps, be aware of the specific assumptions of each theory concerning the area of your investigation. If you overlook a crucial assumption, your conclusions may be rejected from a theoretical standpoint. An exemplary paper (de Gardelle *et al.* 2009) employed masking in a partial report paradigm and interpreted the results as evidence against RP. According to RP, however, visual masking disturbs unreported weak experiences. Therefore, Block (2011) dismissed the interpretation. If de Gardelle *et al*. had employed a paradigm more similar to the original Sperling paradigm, that would have given them a stronger case.

In the previous sections, we discussed how to perform meaningful comparisons, but we have not touched upon the areas in which such comparisons may be most important. Specifying this would aid the work needed for Step 3: defining essential positions. Before comparing theories, it is essential to identify the topic of comparison. Should it be a specific empirical effect that a consciousness theory should explain (Doerig *et al.* 2020) or a more general set of features we associate with consciousness?

### Areas of comparison for empirical theories of consciousness

In a recent paper, Doerig *et al.* (2020) laid out four criteria that they deemed each consciousness theory should address. As an example, they propose *the unfolding argument*. Theories proposing that specific arrangements of neurons are necessary for consciousness should explain their reasoning. For instance, it is a mathematical fact that feedforward networks can mimic the input–output function of recurrent networks (Doerig et al. 2020). Therefore, any theory stating that recurrent processes are necessary for consciousness needs to address why this is indeed the case.

In the published responses to Doerig *et al.* (2020), the authors’ diagnosis concerning the multitude of theories was generally acknowledged. In contrast, there was disagreement with their criteria and assumptions. Most notably, some commentators pointed out that Doerig *et al*. focused only on a functionalist approach to consciousness (Fahrenfort and van Gaal 2020) and that their criteria were generally not atheoretical (Naccache 2020). Considering the shortcomings of Doerig *et al.* approach (2020), we sought inspiration in other fields of cognitive science.

The theoretical diversity seen in consciousness research is not unique and as Northoff and Lamme (2020) were inspired by theory comparison in physics and biology, we believe an inspiration can be found in cognitive science. For example, several of the issues discussed so far were addressed in a recent paper by WM researchers (Oberauer *et al.* 2018). The paper, written by authors of several often-opposing theories, points out the plethora of theories and empirical results concerning WM. They further discuss that each theory only explains a subset of the empirical results that are most often observed in the field: which of the results each theory explains often seem arbitrary. To reduce this idiosyncrasy, they surveyed their colleagues about which empirical effects were most relevant to WM and were most robustly replicated across diverse paradigms. From this, they created a series of benchmarks on which there was a wide agreement in the field (but see: Logie 2018; Vandierendonck 2018).

While the approach of Oberauer *et al*. seems reasonable for WM, it is less obvious if it can also work for consciousness, especially because it may not be possible to define consciousness in purely functional terms, which is how WM is currently characterized. Pinto and Stein (2020, 2) write: ‘[C]onsciousness science faces a unique problem. Unlike nearly all other scientific problems, the aim is not to explain a certain observable function but a first-person phenomenon’. Perhaps agreements on benchmark empirical findings are rendered difficult due to the uniqueness of the consciousness phenomenon?

Instead of focusing on specific empirical phenomena for theory comparison, we can move to a higher level of abstraction. Continuing the analogy with the WM field, Oberauer *et al.* associated benchmarks with features of WM. For example, the effect of set size on accuracy is associated with the core feature of WM: the ability to hold limited amounts of information for current processing, which is defined as memory capacity.

Maybe instead of finding empirical benchmarks that everyone would agree to be crucial for defining consciousness, we could start by defining the core features of consciousness. For purely exemplary reasons, these may include (i) *qualitativecharacter*—‘what it is like’; (ii) *phenomenalstructure*—intentional and representational organization, unity; (iii) *self-perspectiveorganization*—the role of the subject and (iv) *dynamicflow* (based on Van Gulick 2018).

Suppose we could decide which features of consciousness are crucial in describing this phenomenon. In that case, we could move the existing theories closer together. Identifying the criteria (be they empirical phenomena or defining features) that a theory of consciousness should account for is good ground preparation for comparing theories. It could help narrow down the process by focusing on the defining features (as we did in Table 1) instead of all the possible predictions that the theories of interest make. A set of the essential positions a theory of consciousness should account for would substantially aid with comparing theories (see Step 3 in the description of our approach). Additionally, it would help to determine whether a theory has any ‘blank spots’ regarding what is considered a defining feature for a theory of consciousness. For instance, one theory could claim that attention is essential to consciousness. In contrast, another theory might not even use the term.

Importantly, decisions on such core features should be made by the community rather than by individual research groups, as the latter may view things through a specific theoretical lens. For instance, one could argue that the five axioms from IIT are an attempt to do exactly what we ask for. But as they have been criticized, we came up with some examples that seem less controversial to us.

For these reasons, the few features we suggest should be seen as merely the starting point for a wider discussion. Such discussions might be undertaken as part of a separate event at a conference, a dedicated grant proposal focused on theoretical comparison, a workshop, a questionnaire or as a collaboration on a paper similar to one recently written by visual metacognition researchers (Rahnev *et al.* 2021).

## Discussion

The field of consciousness research seems flourishing in terms of the number of publications and conferences. However, contradictory theories are often available, and they are often not related to each other. Are the critics (Irvine 2014; Doerig *et al.* 2020) correct in their diagnosis that researchers often find evidence to support their theory of choice rather than progress the field? We did not find evidence to the contrary with our brief review. For IIT and GWT, which are two of the most widely supported theories, 93% of the papers published in the last decade did not even mention the opposite theory. We have proposed a way to address this siloing: employing multiple hypotheses based on different theories.

Based on experience from another field of cognitive science, directly comparing competing views can help to clarify theoretical views and reach some points of consensus. In the field of WM, adversarial collaborations have at least seemed to bring competing theories closer together (Cowan *et al.* 2020). This is not a panacea, but it may well be the right direction for the consciousness field, especially if we remember we share a similar issue with WM. There are too many theories that are developing in isolation from each other.

SI could be an approach for individual researchers to make theories relate more directly to each other, especially if the six theoretical steps we offer are used. The steps may likely be improved upon with further iterations. Still, they worked for us and, given their general character, will likely work in other cases too.

Of the steps we propose, translating theories into ‘the same language’ is crucial. Empirical tests of hypotheses would be further aided if more similar terms were natively used in different theories. However, to do this will require wide discussions in the field about what a theory of consciousness is supposed to explain. In WM, a broad representation of theorists aimed to establish which empirical results a theory of WM should explain (Oberauer *et al.* 2018). The discussions in our field should perhaps be even more general and ideally happen amongst researchers with diverging views. We have already seen suggestions from a few authors regarding the crucial criteria that a consciousness theory should address (Doerig *et al.* 2020). They were lauded for their attempt by other researchers, who nonetheless often disagreed with the specifics (Fahrenfort and van Gaal 2020; Naccache 2020). We need to aim for consensus on the specific criteria or features that a consciousness theory should account for to stimulate progress in our field.

Suppose theories can converge on terminology and agree on the most relevant features. In that case, this could even lead to fewer theories in the long run. Imagine, for instance, that representatives of *Theory X* agree that a certain feature matters to consciousness. In that case, it would be difficult for them to explain away critical observations subsequently and say that they are not important to consciousness after all. However, the immediate goal of comparing theories is to create clarity rather than to dismiss theories. The latter may well not be possible in a single experiment in any final sense according to the *Duhem–Quine thesis*.

There may be many reasons why formal comparisons are rarely seen in our field today. Some could be personal reservations, like attachment to a single theory, fear of ‘sticking your neck out’ or the fact that more work needs to be done in the preparatory stages. With the growing popularity of study preregistration, researchers are already getting used to spending more time in this stage. As with preregistration, we believe that the issues related to the process will often be outweighed by the benefits. We have recently seen steps toward adversarial collaborations in our field with the high-profile Templeton project (Reardon 2019). Leading researchers from both IIT and GNW have reportedly agreed on predictions, but, to the best of our knowledge, they have not yet disclosed the details of their predictions. We hope this project will be fruitful, harmonize terminology and inspire other researchers to compare theories—even researchers without a high profile within a theory.

It is worth restating that we do not believe that formal comparisons and wide discussions are the only approaches that can progress our field. Exploratory research and construction of individual theories are still important parts of a mature research field. We do, however, believe that formal comparisons and wide discussions should be employed more often than they currently are.

## Data Availability

Figure 1 was based on publicly available data from Google Scholar. We are also happy to share it upon request.
